# Ultrathin niobium nanofilms on fiber optical tapers – a new route towards low-loss hybrid plasmonic modes

**DOI:** 10.1038/srep17060

**Published:** 2015-11-23

**Authors:** Torsten Wieduwilt, Alessandro Tuniz, Sven Linzen, Sebastian Goerke, Jan Dellith, Uwe Hübner, Markus A. Schmidt

**Affiliations:** 1 Leibniz Institute of Photonic Technology e.V., Albert-Einstein-Str. 9, 07745 Jena, Germany; 2 Otto Schott Institute of Material Research, Fraunhoferstr.6, Friedrich-Schiller-University, 07743 Jena, Germany; 3 Abbe Center of Photonics, Friedrich-Schiller-University, Max-Wien-Platz 1, 07743 Jena, Germany

## Abstract

Due to the ongoing improvement in nanostructuring technology, ultrathin metallic nanofilms have recently gained substantial attention in plasmonics, e.g. as building blocks of metasurfaces. Typically, noble metals such as silver or gold are the materials of choice, due to their excellent optical properties, however they also possess some intrinsic disadvantages. Here, we introduce niobium nanofilms (~10 nm thickness) as an alternate plasmonic platform. We demonstrate functionality by depositing a niobium nanofilm on a plasmonic fiber taper, and observe a dielectric-loaded niobium surface-plasmon excitation for the first time, with a modal attenuation of only 3–4 dB/mm in aqueous environment and a refractive index sensitivity up to 15 μm/RIU if the analyte index exceeds 1.42. We show that the niobium nanofilm possesses bulk optical properties, is continuous, homogenous, and inert against any environmental influence, thus possessing several superior properties compared to noble metal nanofilms. These results demonstrate that ultrathin niobium nanofilms can serve as a new platform for biomedical diagnostics, superconducting photonics, ultrathin metasurfaces or new types of optoelectronic devices.

Ultrathin metallic nanolayers attract substantial attention in photonics research, allowing to manipulate light in unprecedented ways via engineered oscillating charge distributions, i.e. plasmonic resonances. For instance, a combination of metallic and high refractive index (RI) layers of thicknesses below 10 nm has been used to uniquely tailor the reflection properties of optical coatings by selectively absorbing various frequencies of the incident light, with promising applications in optoelectronics and green photonics^1^. Another example is given by so-called metasurfaces^2^,^3^, whereby nanostructured elements composed of ultrathin noble metal films of deep subwavelength lateral dimensions form artificial dipoles with engineered phase delay and designed scattering directionality, giving rise to unique optical functionality such as the generation of sophisticated states of light, e.g. orbital angular momentum states^4^,^5^, photonic Spin Hall Effect^6^, ultrathin flat lenses^7^ or extra-strong focusing of light^8^.

One of the most important optical excitations on ultrathin metallic films are surface plasmon polaritons (SPPs), which are hybrid plasmonic-photonic states existing at metal-dielectric interfaces, relying on an intense interaction of an electromagnetic wave with the electron ensemble of the metal^9^,^10^. In contrast to dielectric waveguides, these excitations are capable of confining the propagating modes laterally to deep nanoscale dimensions, hence making them attractive for sensing, e.g. with applications in bio-chemical reaction monitoring^11^,^12^ or in disease diagnostics on the molecular level^13^,^14^. The key functionality of this type of nanosensor is its high sensitivity to small RI-changes within a nanometer environment adjacent to the metal layer^10^. Moreover, SPPs are promising candidates for substantially downscaling current optoelectronic components^9^,^15^, with the vision of integrating nanoelectronics and nanophotonics on an ultra-compact platform with an unprecedentedly small footprint.

Almost all current plasmonic devices rely on noble metals, which reveal comparably low optical loss in the visible (VIS) spectral domain. From the optical point of view, silver is one of the best candidates for nanoscale plasmonics, since it has no interband transitions in the VIS, a plasma wavelength deep in the ultraviolet (UV, plasma wavelength 137 nm) and low optical damping, hence representing a pure Drude metal^16^. However, silver presents the severe disadvantage of being corrosive, leading to the immediate formation of brittle oxide layers even in aqueous solution, which has prevented its widespread application. Aluminum would be an attractive alternative due to its intrinsic high electron density^17^ but reveals similar corrosion problems, presents slightly higher damping, and is difficult to structure. Many of the practically relevant devices rely on gold as the plasmonic material, which despite possessing relatively high optical damping in the VIS due to strong interband transitions^16^,^18^, is intrinsically inert, long-term stable, biocompatible, and easy-to-structure. However, reducing the thickness of gold films is unfeasible, as the growing process of gold films is seed-initiated, leading to discontinuous films (so-called “island growth”) when the thickness falls below ten nanometers^19^,^20^. Moreover, the adhesion of gold on glass substrates is rather poor, imposing the need for additional adhesion layers (e.g. Ti or Cr) between the dielectric and the metal, which can deteriorate the optical properties of the device, particularly if the film thickness falls below ten nanometers, as adhesion layers typically impose more optical loss. This additional deposition step often requires that the sample be transferred into a different deposition apparatus, resulting in oxide formation on the adhesion layer (especially in the case of Ti), thus significantly altering or even degrading the optical properties of the gold. A comparably new approach to improve gold film properties relies on chemically growing single crystal gold films rather than using gas-based deposition techniques^21^,^22^, which yields better film qualities when approaching the sub-ten nanometer level.

Here, we introduce ultrathin niobium films as a novel plasmonic material, revealing intrinsically better film properties in terms of film continuity, mechanical stability and chemical resistivity, when compared to conventional noble metals. The use of niobium films is widespread in current superconducting thin film applications, and in high-sensitive superconducting quantum interference devices (SQUIDs) and superconductor electronics^23^. As a result, the films have been entirely optimized with respect to their superconducting, i.e. electrical properties, demanding precise control of the film thickness and quality on the sub-nanometer level. Moreover, the progress in the field of integrated SQUID technology has substantially driven the development of nanostructured ultrathin niobium films, currently reaching lateral feature sizes of tens of nanometers^24^,^25^,^26^,^27^. In contrast to gold, niobium films reveal substantially better adhesion to glass, in particular to silica, leading to solid metallic films which cannot be mechanically removed from glass surfaces without destroying the actual sample surface. This is especially relevant in the framework of ultrathin layers, as no additional adhesion layer is required as in the case of gold. Continuous niobium films are already obtained below a film thickness of 10 nm–at which traditional gold deposition approaches lead to isolated gold islands rather than compact films^19^,^20^. After deposition, niobium naturally forms a several-atom thick oxide protection layer, keeping the metallic film separated from the environment^28^. Here we show that this protection layer is so effective that the niobium remains plasmonically inert even if being exposed to aqueous solutions, making such ultrathin films particularly attractive for plasmon-mediated biosensing applications. Furthermore, oxide film formation can be controlled and even enforced on the nanometer level via electrolytic passivation, i.e. anodization^29^; since the adhesion of the oxide layer to the niobium film is extremely strong, this leads to an intrinsically robust multilayer with predefined thickness, thus making additional film deposition steps obsolete.

## Results

### Plasmonic properties of ultrathin niobium films

Even though ultrathin niobium films reveal superior mechanical, chemical and nanostructuring properties, the immediate question from a plasmonics point of view is: Do ultrathin niobium films support SPPs and how do their properties compare to state-of-the-art materials such as noble metals?

To address this, a performance parameter comparison of the SPPs supported by metallic nanofilms (i.e. long-range (LR) and short-range (SR) SPPs) are presented here for three different metals (details regarding film plasmons (e.g., field profiles) can be found in Ref. 30). The most important property of the SR-mode is the strong lateral confinement of the electromagnetic field to the metal. The SR-plasmon performance can be quantified via a confinement figure-of-merit *C*_FOM_ parameter relying on the penetration depth *δ* of the magnetic field into the dielectric, leading to:


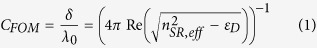


where *λ*_0_ is the wavelength in vacuum, *n*_SR,eff_ is the complex effective mode index of the SR-SPP, and *ε*_D_ is the dielectric function of silica. Here, we assume that the metal film is symmetrically sandwiched between two semi-infinite layers of silica, with material dispersion given in the work of Fleming^31^. A deep subwavelength confinement is reached once the penetration depth is substantially smaller than the operation wavelength, i.e. *δ* ≪ *λ*_0_. The complex mode index is obtained by numerically solving the corresponding transcendental dispersion equations^30^. The SR-modes are generally important for applications demanding strong field confinement, such as near-field scanning microscopy^32^,^33^.

LR-plasmons propagate over distances orders of magnitude longer, at the expense of less confinement. The attenuation of these modes is directly related to the fraction of electromagnetic field in the metal^34^. The corresponding attenuation figure-of-merit parameter *A*_FOM_ can be defined accordingly via the propagation length of the LR-SPP, which is related with the imaginary part of the effective mode index via


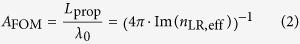


Here, *L*_prop_ is the propagation length of the LR-SPP with respect to intensity (defined as the length at which the intensity drops by a factor of 1/e) and *n*_LR,eff_ is the (complex) effective index of the LR-mode.

**Figure 1:**
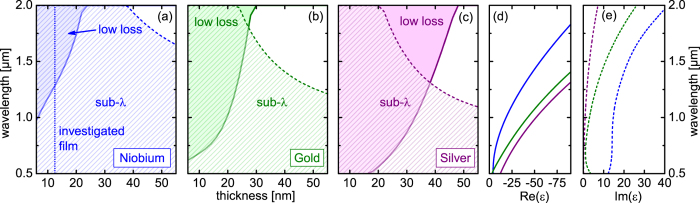
Comparison of confinement and loss properties of surface plasmon polaritons propagating on niobium (a, blue diagram), gold (b, green diagram) and silver (b, purple diagram), assuming that the metal films are sandwiched between two semi-infinite silica layers. In the three left-handed performances maps (**a**–**c**), the hatched areas refer the regions of subwavelength confinement (*δ* < *λ*_0_/2) of the SR-SPP mode. The filled colored regions indicate the domain of long-range propagation (*L*_abs_ > 1000 *λ*_0_, long-range regime) of the LR-plasmon. The vertical dotted blue line in Fig. 1a indicates the thickness of the niobium film used in the experiments. The properties of the complex dielectric functions of the three discussed materials (Nb: blue, Au: green, Ag: purple) are shown in the two right-handed diagrams ((**d**): real parts of dielectric function (solid lines), (**e**) corresponding imaginary parts (dashed curves)).

To understand the plasmonic performance of niobium films and to place the results in the context of state-of-the-art plasmonic materials, the dependence of both these figure-of-merit parameters on film thickness and wavelength have been determined for niobium, gold and silver (Fig. 1a–c). To obtain a quantitative comparison between these three metals, fixed practical values of the figure-of-merit parameters are considered, allowing a comparison of the materials on the basis of performance maps (Fig. 1a–c).

For the SR-plasmons, it is of practical interest when the penetration depth is about half of the operation wavelength, leading to *C*_FOM_ = *δ/λ*_0_ < 0.5 (hashed areas in Fig. 1a–c). In the case of LR-SPPs, a significant propagation length is achieved if the plasmon travels for more than five hundred wavelengths, resulting in *A*_FOM_ = *L*_prop_*/λ*_0_ > 1000 (solid filled area in Fig. 1a–c). The choice of this value for *A*_FOM_ is rather arbitrary, but relates to the practically relevant case of a propagation length of the orders of millimeters (e.g., at a wavelength of 1 μm, *A*_FOM_ = 1000 corresponds to a propagation length of one millimeter).

Propagation across a millimeter distance can be reached in the case of niobium when the film thickness falls below 20 nm (Fig. 1a). Comparing the three *A*_FOM_-parameters shows that the low-loss area in the niobium performance map is reduced, which is a result of the imaginary part of the dielectric function of niobium (Im(*ε*_Nb_), blue dashed line in Fig. 1e) being larger than that of gold and silver (Im(*ε*_Au_) and Im(*ε*_Ag_), green and purple dashed lines in Fig. 1e) for all investigated wavelengths. At first glance, this might suggest a less favorable performance, however this can be compensated by the fact that high-quality ultrathin niobium films can be straightforwardly fabricated at thicknesses where the deposition of gold and silver films becomes problematic (e.g., due to island growth^19^,^20^). It is interesting to note that the actual performance of the gold-LR-SPP is only slightly better than that of the corresponding niobium plasmon.

A reverse situation is observed when comparing the confinement properties of the SR-plasmons (*C*_FOM_-parameter): Here, the Nb-SPP shows a substantially better confinement than gold and silver for all relevant wavelengths (note that the hashed area in the performance map of Fig. 1 is greatest in the case of niobium), which is associated with the relatively large magnitude of Im(*ε*_Nb_), leading to an increase of the imaginary part of *n*_eff_. As a consequence, the transverse wave vector is increased, and *C*_FOM_ is reduced. This finding represents a substantial result, as it suggests that an improved confinement performance of the SR-SPP requires a larger the material attenuation, which to some extent is counter-intuitive.

Note that we have measured the dielectric function of niobium on a test film having a thickness of 300 nm, whereas bulk literature values for *ε*_Ag_ and *ε*_Al_ have been used^16^. Our experiments clearly show that the measured values of *ε*_Nb_ are in perfect agreement with the simulations, showing that ultrathin niobium films possess bulk optical properties. This robustness of the optical properties when reducing niobium film thickness is intrinsic to the material itself. The situation is different for thin gold films, which changes its characteristics when the thickness falls below 10 nm, as reported by Kossoy *et al.*^21^.

### Hybrid plasmonic modes niobium optical fiber tapers

**Figure 2:**
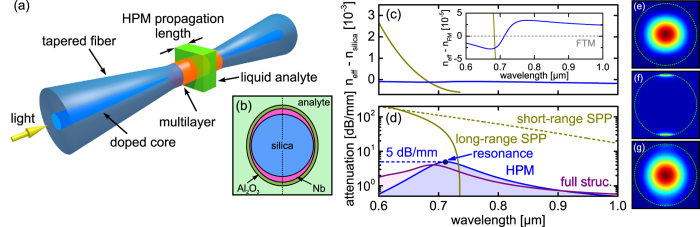
Excitation of propagating plasmons on ultrathin niobium films using a multilayer on a fiber optical taper. (**a**) Sketch of the plasmonic fiber taper with the section of the multilayer structure. (**b**) Schematic of the cross section of the taper within the plasmonic section. Different colors indicate the materials used (blue: silica, magenta: ultrathin niobium film, dark green: Al_2_O_3_ layer, light green: liquid analyte). The dashed black vertical line refers to the cross section which is used for the planar multilayer simulations. (**c**) Normalized effective index dispersion of the different modes of the systems, calculated using the planar symmetric multilayer model and considering TM-polarization. The blue curve refers to the hybrid plasmonic mode (even TM_01_-mode), and the dark yellow curve is the long-range SPP of the asymmetrically embedded film (no mirror symmetry in the silica assumed, SPP is evanescent in silica and analyte). The inset is a close-up view of the section at which the resonance occurs (to emphasize the domain of the resonance, the effective mode indices have been normalized to the dispersion of the fundamental taper mode which has no multilayer). (**d**) Attenuation of the modes plotted in (**c**). The purple curve indicates the modal attenuation of the HPM of the realistic full structure (realistic structure shown in (**b**), assuming maximum and minimum NB film thicknesses of 12.5 nm and 3 nm, respectively) calculated using Finite-Element modeling. For completeness, this plot also includes the attenuation of the SR-SPP (dashed dark yellow line). The right-handed images show the Poynting vector distributions of the dielectric (**e**) and SPP mode (**f**) (both at 600 nm) and the HPM at resonance ((**g**), 690 nm). The dashed green circles indicate the surface of the taper. In all simulations, we assume niobium and Al_2_O_3_ films thicknesses of 12.5 nm and 80 nm, respectively. The analyte is assumed to have negligible loss and dispersion in this spectral domain with a constant index of *n*_a_ = 1.36.

To experimentally investigate the plasmons supported by ultrathin niobium films, we choose an optical fiber taper based approach, which allows exciting the plasmons via the evanescent field of the fundamental taper mode (FTM)^35^. The taper itself is a cylindrical rod of fused silica glass (20 μm diameter) surrounded by two oppositely faced sickle-type layers of ultrathin Niobium (maximum thickness: 12.5 nm, Fig. 2a). The entire taper is coated with a high-index dielectric (Al_2_O_3_, thickness 80 nm) and is embedded in a liquid analyte with a predefined RI. As the radius of the experimentally investigated tapers is comparably large, the surface curvature of the taper can be neglected - see for example Eq. (5) of Ref. 36, which shows that the dispersions of plasmons in metallic wires approach the dispersion of the planar plasmon once the radius is larger than 10 μm at a wavelength of 1 μm. This allows us to approximate the entire plasmonic taper as a planar multilayer which is infinitely extended perpendicular to the propagation and normal film axis (see Methods Section for details). To resemble the Gaussian-type profile and the polarization properties of the FTM, central mirror symmetry at the boundary of the silica is considered, leading to a symmetric cosine-type behavior of the dominant magnetic field inside the silica. This one-dimensional geometry substantially reduces simulation efforts, and propagating plasmons are observed when transverse-magnetic (TM) polarization is considered^30^. From the planar waveguide perspective, the hybrid mode with such symmetry is commonly referred as the even TM_01_-mode. Although it is well known that for increasingly large fiber radii, the fundamental fiber modes are asymptotically TM_01_ modes^37^, we have verified the validity of the approximate planar model by comparing the calculated attenuation value (blue curve in Fig. 2d) with the finite element simulations of the full structure (purple curve in Fig. 2d, structure shown in Fig. 2b). We find that the planar model follows the evolution of the full structure with a slightly red-shifted resonance and with a peak attenuation coefficient which is 20% higher than that of the full structure. As a result, the planar model provides a way to estimate the upper limit of the attenuation of the hybrid mode at resonance (see below), as well as providing a straightforward way to predict the plasmonic resonance positions and analyze the underlying physics.

The basic working principle of this geometry relies on the formation of a hybrid plasmonic-photonic eigenstate (Fig. 2g) when the effective indices of the FTM and the LR-SPP modes become relatively close (Fig. 2c,d). Since we are experimentally targeting analytes with RIs in the interval between 1.33 and 1.44, an additional dielectric layer (here: Al_2_O_3_) is required to ensure that dispersions of the effective indices of FTM and LR-SPP become sufficiently close.

At first glance the ultrathin-film-based plasmonic taper may suggest a typical two waveguide, i.e. directional waveguide coupler situation^38^. This, however, is an insufficient description as the FTM is not evanescent within the metal i.e. there is no evanescent field coupling between the mode on the ultrathin film and the core in the taper core. In the system described here, the incoming FTM (example images of the mode is shown in Fig. 2e) directly excites the two hybrid Eigenstates which are formed when the effective indices of the plasmons and the FTM approach. The coupling efficiency of the individual hybrid supermode at a fixed wavelength is determined by the overlap of the FTM with the respective mode. The position at which the spectral distribution of the attenuation peaks is roughly the position at which the dispersion cross (weak coupling domain (Fig. 2c)) or anti-cross (strong coupling domain) and is thus referred here as plasmonic resonance with the resonance wavelength *λ*_R_.

Using the plasmonic fiber approach is intrinsically advantageous over other excitation techniques from various points of view: The direct excitation of the hybrid Eigenstate results in a translationally invariant mode propagation inside the metal coated section, which is particularly advantageous when compared to a coupler geometry. A directional plasmonic coupler requires the coupling length to be substantially shorter than the attenuation length of the isolated SPP, which is typically of the order of tens of micrometers and thus demands extremely strong coupling, which can be difficult to obtain experimentally^39^. Depending on the fraction of magnetic field in the taper and metal, the magnitude of the modal attenuation of the HPM can be tuned to almost any desired value by adjusting the diameter of the taper and the thickness of the metal film. In fact, most of the field is located inside the silica (which is also evident from the small magnitude of the crossing of the effective indices close to the resonance – see inset of Fig. 2c), allowing to reduce the attenuation of the HPM down to the dB/mm level, which is experimentally practical and allows to investigate the properties of the niobium SPPs in great detail. The possibility of adjusting the attenuation down to such low levels is one of the key features of the plasmonic taper approach, as it fundamentally allows investigating materials with comparably high attenuation. Therefore, the taper approach has the intrinsic capability of making more materials accessible for the plasmonics community than using any other method. The high fraction of field in the glass taper also allows very efficient excitation of the HPM, since the electromagnetic fields in plasmonic and uncoated taper sections have comparable lateral distributions. A simple estimation using the fields from the planar multilayer model and an overlap integral approach^40^ shows that the launching efficiency is >93%, which is superior to any of the known excitation techniques in plasmonics such as Kretchmann excitation or grating coupling^41^. In the case of a Kretschmann configuration, prism incidence angles >88° would be necessary to excite a SPP on an ultrathin niobium film. Here we choose niobium and Al_2_O_3_ film thicknesses of 12.5 nm and 80 nm, respectively, to achieve a maximal ratio of peak height vs. peak width (full width at half maximum) at an analyte index of 1.38. This ratio represents the optimal conditions for sensing experiments, which demand on-off transmission ratios of <1% and, from the practical point of view, millimeter interaction lengths.

It is important to note that a dipole-type SPP mode with oppositely-facing lobes is excited (Fig. 1c), since it must possess the same azimuthal order as the exciting FTM, which is in fact an HE_11_ mode with an azimuthal order of unity (*m* = 1). Experimentally, the length the HPM can be adjusted by changing the length of the liquid column of the analyte, which can be easily conducted in the experiment by changing the analyte basin, thus providing a straightforward path to obtaining quantitative figures for the modal attenuation (in fiber-optics terminology, this procedure can be interpreted at “non-destructive cutback method”).

Our ultrathin niobium films are deposited on test glass slides and fiber tapers using DC magnetron sputtering (details can be found in the Methods Section). Continuous and smooth niobium films with an rms roughness of only 1.8 nm at a total film thickness of 12.5 nm have been obtained, whereas the roughness of the uncoated silicon substrate was ∼0.2 nm. The dielectric function of niobium was determined by ellipsometrically measuring the optical constants of optically thick niobium films (thickness 300 nm, the real and imaginary parts of *ε*_Nb_ are shown by the blue curves in Fig. 1d,e and in the Methods Section). Due to oxidation, niobium films automatically form nanometer-thick amorphous oxide layers which act as a protective film^28^. Simulations confirm that the thin oxide layer serves no optical functionality.

The plasmonic fiber tapers consists of a down-tapered commercially available step-index fiber (Nufern HP780) which is single-mode down to a wavelength of approximately 730 nm (outer fiber diameter 125 μm). The total length of the resulting taper was ∼80 mm (using a Vytran GPX 3000 (AMS Technologies)) with a waist length of ∼10 mm (constant diameter section) and a diameter of 20 μm. The lengths of the two transition zones (35 mm) were chosen to avoid higher-order mode excitation, giving rise to adiabatic transitions and a clean fundamental mode in the waist section. It is important to note that in the constant diameter section, the influence of the doped core on the optical properties of the taper is so small that it can be neglected. The mode inside the waist can thus be represented by the fundamental (HE_11_) mode of cylindrical silica strand in air. Two ultrathin niobium films were deposited on two opposite sides of the taper waist leading to a double sickle-type structure (Fig. 2(b), fabrication details can be found in Methods Section). The length of the niobium film closely corresponds to the length of the taper waist. To ensure that the effective indices of FTM and LR-SPP become sufficiently close, an additional Al_2_O_3_ layer was created on the taper. Since an atomic layer deposition process was used, an extremely smooth and high-quality dielectric film (rms roughness ∼0.5 nm) with a constant thickness of 80 nm was concentrically produced around the entire niobium coated taper. The optical constants of that film have been ellipsometrically determined on a suitable planar test sample (details in the Methods Section).

The optical characterization of the plasmonic tapers was conducted using a combination of a supercontinuum light source, sample, and an optical spectrum analyzer (OSA, used resolution: 1 nm, further details can be found in the Methods Section). Sample heating by infrared light was excluded by inserting a water-filled cuvette after the light source, and polarization control was provided by a polarizer before the in-coupling objective. In the experiment, the direction of the input polarization was adjusted such that *λ*_R_ is as large as possible for a given configuration, which corresponds to that polarization state at which an SPP is exited along the direction of largest film thickness (which is the situation illustrated by the black dashed line in Fig. 1b). An additional polarizer was inserted after the output of the taper to increase polarization contrast and great care was taken to prevent polarization cross-coupling (total fiber length 20 cm including the two taper transition regions and the multilayer part in its center). We obtained a polarization ratio of 1:100 (20 dB). Desired analytes (in this case, different RI-liquids) are brought into contact with the taper using suitable Teflon stubs. The length the HPM propagates is adjusted via the length of the liquid covering the taper, which was experimentally established by using different stubs. To remove the spectral characteristics of the optical components (e.g. light source or objectives), all measured transmission curves have been referenced to equal measurements with no analyte surrounding the plasmonic layers, which corresponds to the case of no SPP excitation.

**Figure 3:**
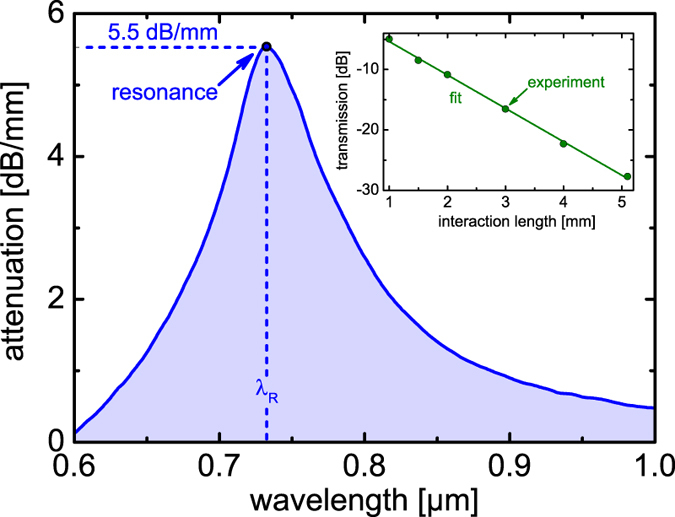
Measured spectral distribution of the modal attenuation of the hybrid plasmonic mode of the ultrathin film based taper in the case of a predefined analyte (Cargille liquid, na = 1.36 at a wavelength of 589 nm). The inset shows the measured transmission values (in logarithmic scale) for different stub lengths (length of the analyte column) at resonance (*λ*_R_ = 732 nm). The straight green line is a linear fit to the experimental data (in dB-scale), with the slope corresponding to the modal attenuation of the propagating hybrid plasmonic mode.

To obtain the modal attenuation of the HPM, several transmission measurements, each with a different analyte length, have been conducted for a fixed analyte index. At each wavelength, the different transmission values (in dB-scale) have been fitted by a linear curve (inset of Fig. 3), giving rise to a quantitative determination of the spectral distribution of the modal attenuation.

The distribution of the RI sensitivity (*S* = Δ*λ*_R_/Δ*n*_a_ with the analyte RI *n*_a_) was determined by choosing different analyte indices (RI-liquids in the range 1.32–1.43) and plotting the resulting measured resonance wavelengths as a function of analyte RI. The resonance wavelengths were determined using a second-order polynomial fitting applied to the minima of the normalized transmission spectra. It is important to mention that the value of *n*_a_ was determined at *λ*_R_ taking into account the material dispersion of the liquid rather than a constant RI value. By numerically performing a derivative of the resulting curve with respect to *n*_a_, the RI-distribution of *S* was obtained. A fixed liquid length of ~1.8 mm was chosen, resulting in sufficiently deep transmission dips to determine the spectral resonance positions.

### Modal attenuation of hybrid plasmonic mode

As discussed, the niobium nanofilm based plasmonic taper creates a HPM at wavelengths where the dispersion of isolated LR-SPP and FTM become close. The formation of the HPM leads to a substantial peak in the spectral distribution of the attenuation of the taper, which can be used to evaluate the quality of the metal film. Using the loss quantification procedure mentioned earlier, we determined the modal attenuation of HPM for one example analyte (Cargille liquid with index 1.36 @ 589 nm), showing a resonance wavelength of 732 nm with an attenuation of 5.5 dB/mm (Fig. 3), which is very close to the simulated value obtained from the planar multilayer model (5 dB/mm, Fig. 2d). A discontinuous, island-type niobium film would be associated with substantial scattering of the HPM at the island boundaries and thus much higher loss. The correspondence between model and experiment indicate that our ultrathin niobium films (thickness 12.5 nm) are of excellent quality, since the simulation presented in Fig. 2 were based on a dielectric function measured using a much thicker film (thickness 300 nm, Fig. 1e,f, see Method section for details). Thus the optical properties of our ultrathin niobium films are identical to those of the corresponding bulk metal, making such films extremely attractive for applications in which nanometer-thick films are required. It is important to note that the modal attenuation mainly depends on Im(*ε*_Nb_), and thus the agreement of simulation and experimental data shows that no additional loss originating from surface roughness is induced at such small thickness and that the film is entirely continuous. From the plasmonics perspective, the measured attenuation figure (5.5 dB/mm) is relatively low^42^,^43^,^44^, making the fiber-plasmonic taper approach interesting if dielectric-loaded SPPs are considered.

It should be noted that the analyte length is an important parameter in plasmonic sensor system using spectral interrogation, because it is preferable to have a deep resonance dip for precisely determining the resonance minimum, i.e. *λ*_R_^45^. This criterion imposes an optimal analyte length for each analyte RI, which can be easily adjusted, given the flexibility of this re-configurable plasmonic device.

### Refractive index sensitivity

**Figure 4:**
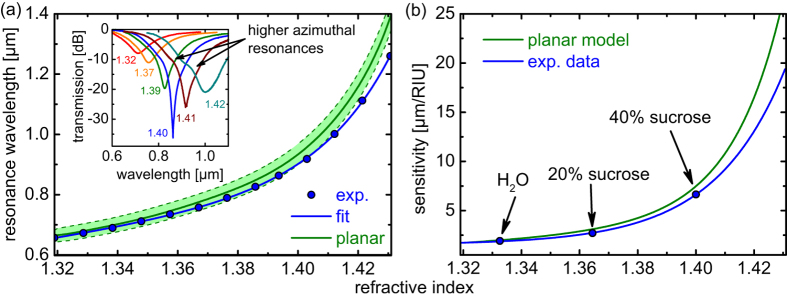
Refractive index properties of the niobium-based plasmonic taper. (**a**) Resonance wavelength as a function of refractive index of the applied liquid. The blue dots refer to the measured resonance wavelength obtained from the dips in the transmission spectra (blue line is a guide-to-the-eye). The green curve shows the results from the planar multilayer model. The shaded green area refers to the potential change in resonance wavelength if a niobium thickness uncertainty of ±1 nm is considered. The inset shows examples of the spectral distribution of selected normalized transmission spectra (values are the RIs of the liquids at *λ*_0_ = 589 nm according to the labeling of the used Cargille analytes). (**b**) Refractive index distribution of the sensitivity of the experimental data (blue) and of the planar multilayer waveguide model (green). The blue dot indicates the sensitivity value at the index of water.

Determining the sensitivity requires precise knowledge of the dependence of the resonance wavelength with respect to the RI of the analyte (inset in Fig. 4a), which was determined here by measuring *λ*_R_ for various analytes. The resonance wavelength shifts towards longer wavelengths as *n*_a_ increases (blue dots and blue line in Fig. 4a), which is a result of the increasing interaction of FTM and LR-SPP and the increased field penetration of the HPM into the analyte. It is important to note that the Niobium system allows access to a spectral domain ranging from visible wavelengths to the near infrared, which makes our system attractive for sensing a great variety of liquids over a broad range of RI-intervals, from 1.32 to 1.43.

Simulations using the planar multilayer model confirm the observed evolution of an increasing *λ*_R_ towards larger values of *n*_a_ (green solid line in Fig. 4a) and agree with the experimental values for all considered RIs to a very high degree. To account for the uncertainties in the measurement of the thickness of the niobium film, imposed by the spatial resolution of the scanning electron microscopy image, we included a possible thickness variation of ±1 nm (light green area in Fig. 4a). Since the resonance wavelength itself is most sensitive to Re(*ε*_Nb_), the good agreement between experiment and theory provides further confirmation that the niobium film indeed possesses bulk optical properties. It needs to be pointed out that for RI larger than 1.40, the system changes from a weakly coupled situation to a strongly coupled system, in which avoided crossing of the FTM and the LR-SPP are observed, which makes the determination of the resonance positions in the calculated attenuation spectrum rather complicated. A precise study of the strongly coupled situation is out of the scope of this contribution and will be considered in a later study.

The modal attenuation of the HPM at *λ*_R_ increases towards larger values of the analyte refractive index, which results from the increasing interaction of FTM and LR-SPP (inset of Fig. 4a). As mentioned earlier, if *n*_a_ > 1.40, the system changes into a strongly coupled situation, leading to a spectral broadening of the transmission dips. Additional short wavelength dips are observed for *n*_a_ > 1.42, which are caused by exciting plasmon modes of higher azimuthal order (*m* > 1)^46^,^47^.

The experimental distribution of the sensitivity rises with increasing analyte refractive index and again agrees well with the simulations (Fig. 4b). Very large sensitivity values above 10 μm/RIU are obtained if *n*_a_ > 1.4, which is an important domain for liquids such as higher n-alkanes, alkanol/n-alkane dispersions or highly concentrated sucrose and sodium chloride solutions. In the refractive index range of aqueous solutions (*n*_a_ = 1.33) the sensitivity of our system is 1.88 μm/RIU, which lies within the range of typical plasmonic and fiber-sensor system^48^,^49^,^50^ and is sufficient for many biomedical and biochemical applications.

## Discussion

In this contribution we have investigated for the first time ultrathin niobium films (thickness 12.5 nm) with respect to their plasmonic properties. Beside an in-depth ellipsometrical characterization and a direct performance comparison to plasmons on noble metals, we uniquely excite niobium plasmons using a flexible optical fiber taper approach. We observe a low-loss hybrid plasmonic mode of modal attenuation of only 5.5 dB/mm (*n*_a_ = 1.36), which represent one of the lowest figures reported, especially if materials with comparably high optical damping are considered. By measuring the modal attenuation and the refractive index sensitivity of that hybrid mode we found that the ultrathin niobium films have the same dielectric function as their bulk counterparts, clearly indicating the exceptional quality of ultrathin niobium. The films are protected by a ~2 nm thick oxide layer after deposition, which is optically negligible but improves the mechanical long-term stability of the film, making it inert against any environmental influence. The films show substantially better adhesion to glass surfaces than noble metals, circumventing the need for additional adhesion layers.

As a consequence of these unique properties, ultrathin niobium films in general represent a promising alterative to noble metals, with several advantageous properties compared to noble metals traditionally used in plasmonics. Our results indicate that this material system reveals no degradation in aqueous solution, providing the base for promising application in biosensing and molecular disease diagnostics. Moreover, niobium is the current workhorse of superconductor industry due to its high critical temperature and, as a result, ultrathin niobium films may represent a promising material for optoelectronics on a plasmon-polariton type approach^51^. Niobium films can be nanostructured in the same fashion as silver without presenting degradation, indicating promising application as ultrathin metasurfaces^2^,^3^ or fibre-based metamaterials^52^,^53^.

## Methods

### Fabrication of the ultrathin niobium film

The deposition of the niobium films was carried out by DC magnetron sputtering using an Alcatel SCM 650 sputtering system with a base pressure of 2·10^–8^ mbar. The niobium target had a diameter of 6″ inches and a purity of 99.95%. To ensure that only the waist of the fiber taper was coated during deposition, the fiber was clamped between two small aluminum plates containing an opening at the waist location. By water cooling the chuck, the fiber was held close to room temperature during film deposition. Adjusting the target-to-substrate distance and using a deposition rate of ∼22 nm per minute at an argon pressure of 0.01 mbar allowed nearly stress-free film growth. After depositing the ultrathin film on one side of the taper, the fiber was rotated along its symmetry axis by 180° within a short break of the vacuum to coat a similar film on the opposite side of the taper. This procedure led to two oppositely facing sickle-type niobium films with maximum thicknesses of (12.5 ± 1.0) nm. In order to improve the adhesive strength of the niobium film to the fiber surface, each fiber side was in-situ cleaned before film deposition by a sputter etch process using argon ions. The roughness of all produced films has been quantified by an atomic-force-microscopy (Autoprobe M5, Park Scientific Instruments).

### Fabrication of the aluminium oxide film

The deposition of the aluminum oxide layers was carried out using plasma-enhanced atomic layer deposition (PEALD) with TMA (Trimethylaluminium) as precursor and oxygen as reactant gas. We used an OpAl ALD machine with plasma option (Oxford Plasma Technology). One ALD cycle consists of the following sequence: injection of TMA precursor, followed by Ar/N_2_-purge, oxygen plasma and finally Ar/N_2_-purge. The growth rate of the aluminum oxide film at a temperature of 30 °C was ∼0.18 nm per cycle, thus 445 cycles were required to establish a film thickness of 80 nm, giving rise to a total deposition time of 2 hours and 45 min. The pressure during the deposition was ∼0.35 mbar.

### Determination of the dielectric functions using ellipsometry

**Figure 5:**
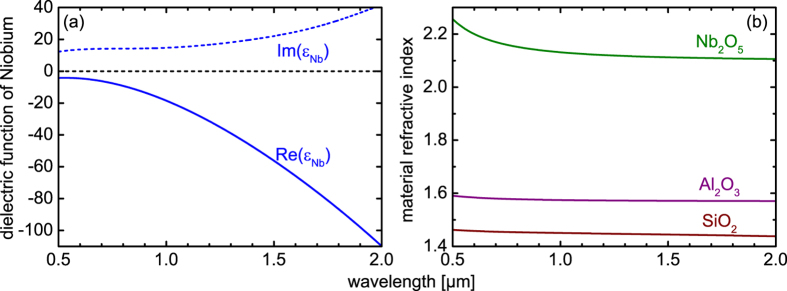
Spectral distribution of the relevant optical quantities of the materials involved. (**a**) Dielectric function of the niobium (measured using 300 nm thick film, solid (dashed) line: real (imaginary) part of *ε*_Nb_). (**b**) Material dispersion of the three used dielectric materials (green: niobium oxide, purple: aluminum oxide, brown: fused silica). Since all three dielectrics have very low absorption in the spectral interval relevant here, the absorption coefficient has not been plotted.

The dielectric functions of niobium, niobium oxide and aluminum oxide were ellipsometrically determined between 500 nm and 1.5 μm using a variable-angle spectroscopic ellipsometer (Sentech, SE850). To measure *ε*_Nb_, a 300 nm thick film was deposited on a silicon wafer, which had an intrinsic flat surface (rms roughness 0.5 nm, Fig. 5a). The thickness of the niobium film was chosen such that it transmits no light in the spectral interval of interest, making the analysis of the *ψ*Δ-data straightforward, since no substrate contribution needs to be taken into account. In case of aluminum oxide, we determined *ε*_Al2O3_ on a 80 nm thick film again on a silicon wafer and used the Tauc-Lorentz (TL) model^54^ for fitting the measured data (Fig. 5b). The imaginary part of the complex dielectric function of a single Lorentz oscillator is given by


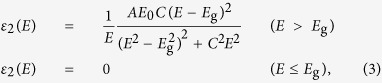


where *E*_g_ is the optical band gap energy, *E*_0_ the peak position of the Lorentz oscillator, *C* the broadening parameter of the Lorentz oscillator and *A* the transition amplitude. The real part of the dielectric function is obtained by using Kramers-Kronig relation:


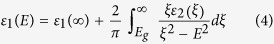


This integral can be equated exactly (including dielectric constant in the high-frequency limit *ε*_1_(*∞*) which is a fitting constant). The final expression is not shown here and can be found in Jellison *et al.*^54^. The TL-model is a valid assumption for *ε*_Al2O3_ in the spectral interval of interest since the films have no significant absorption features for *λ*_0_ > 400 nm. In a similar way we determined the dielectric function of the niobium oxide by anodizing the niobium film and analyzing it ellipsometrically (Fig. 5).

Table 1 shows the fitted parameters of the Tauc-Lorentz model (Eqs. (3) and (4)) for Al_2_O_3_ and Nb_2_O_5_, respectively.

### Experimental transmission setup

**Figure 6:**
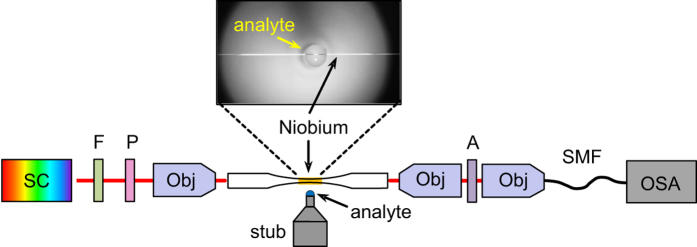
Schematic of the transmission setup used to measure the optical response of the ultrathin film-based plasmonic taper: SC: supercontinuum source, F: water-based filter for removing the infrared light, P: input polarizer, A: analyzer, Obj: microscope objective, SMF: single mode fiber, OSA: optical spectrum analyzer.

To measure the transmission of the plasmonic fiber tapers we used the following experimental setup: Light from a supercontinuum source SC (Fianium SC-450-2, bandwidth: 400 nm to 1.8 μm) was used as the illumination source (Fig. 6). The coupling into and out of the entire fiber was realized by using microscope objectives (Nikon CF Plan 50x/0.55WD 8.7). The spectral distribution of the transmitted light intensity was measured by an OSA (Ando, AQ6315A) which was set to a spectral resolution of 1 nm. To prevent film heating, a 1-cm cuvette filled with water acting as IR blocking filter was inserted just after the output of the light source to avoid absorption of infrared light. A polarizing sheet (foil polarizer, CODIXX model colorPol VISIR) was inserted into the beam path at the input side to ensure that a controlled linear polarization state reaches the sample. Since the taper consists of non-polarization maintaining fiber, the entire sample (total length: 20 cm) was kept as straight as possible to prevent cross-polarization and intermodal coupling. Using an additional polarizer at the output led to a polarization ratio of 1:100 (20 dB), which is sufficient for all measurements presented.

Commercially available RI-liquids (Cargille, Laboratories, Inc.) with indices in the range between 1.32 and 1.43 (nominal values corresponding to a wavelength of 589 nm at a temperature of 25 °C) were used for the determination of RI-sensitivity and modal attenuation. Different analyte lengths were provided by using home-made Teflon stubs with various diameters.

### Model for simulating SPP

**Figure 7:**
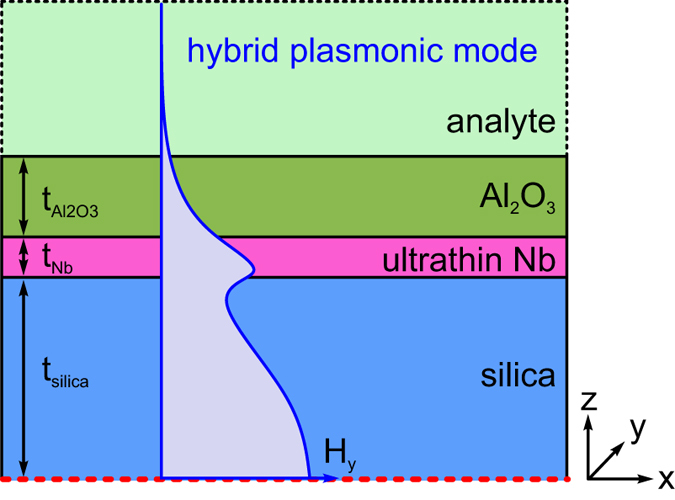
Sequence of the materials used in the planar multilayer model employed to analyze the properties of the modes within the plasmonic taper section. The various colors refer to the different materials involved (dark blue: silica (*t*_silica_ = 10 μm), magenta: ultrathin niobium film (*t*_Nb_ = 12.5 nm), dark green: Al_2_O_3_ (*t*_Al2O3_ = 80 nm), light green: liquid analyte). The blue curve is a schematic representation of the magnetic field components (*H*_y_) of the TM-polarized hybrid plasmonic mode (even TM_01_-mode, mode amplitude and shape of the curve not to scale). The propagation direction is along the *x*-axis, and the waveguide is translational invariant along the *y*-axis. The dashed red line indicates the symmetry plane at which the boundary condition ∂*H*_y_/∂*z* = 0 is applied.

The calculations presented here are based on a planar multilayer model assuming translational invariance along the *y*-direction (Fig. 7). Propagating plasmons are excited in this structure when TM-polarization is considered, leading to three non-zero electromagnetic field components (here: *H*_y_, *E*_x_, *E*_z_). It is assumed that the propagation direction and the layer sequence are along the *x* and *z*-axes, respectively, whereas the outermost layer (analyte, light green in Fig. 7) is extending to infinity. To reproduce the situation of the constant diameter section (metal coated section) of the taper, it is assumed that the mode profile of the magnetic field component (*H*_y_-component) has mirror symmetry along the *x*-axis at *z* = 0 (dashed red horizontal line in Fig. 7), imposing to a von-Neumann type boundary condition (∂*H*_y_/∂*z* = 0 at *z* = 0). The application of this particular condition is justified by the fact that only the fundamental taper mode (HE_11_) is excited in the constant diameter section as adiabatic taper transitions have been used here. In planar waveguide terminology, the mode with this type of symmetry and polarization is commonly referred as even TM_01_ mode. Surface roughness is not included into the model, which is *a posteriori* justified by the match of loss figures and resonance positions of experiment and simulation.

The result of the simulation is the complex refractive index, allowing to directly calculate the modal attenuation using *γ*_M_ = Im(*n*_eff_)·2π/*λ*_0_ with the effective mode index *n*_eff_ and the vacuum wavelength *λ*_0_.

## Additional Information

**How to cite this article**: Wieduwilt, T. *et al.* Ultrathin niobium nanofilms on fiber optical tapers - a new route towards low-loss hybrid plasmonic modes. *Sci. Rep.* **5**, 17060; doi: 10.1038/srep17060 (2015).

## Figures and Tables

**Table 1:** Parameters obtained from fitting the experimental ellipsometric data of the Al_2_O_3_ and Nb_2_O_5_ films with the Tauc-Lorentz model.

	*ε*_1_(∞)	*E*_g_[eV]	*E*_0_[eV]	*A*[eV]	*C*[eV]
Nb_2_O_5_	1.063	3.488	4.006	350.9	3.067
Al_2_O_3_	1.002	6.485	8.983	190.5	6.468
